# Triplet–Triplet Annihilation Upconversion Is Impeded in Liposomes that Prevent Sensitizer and Annihilator Co-Confinement

**DOI:** 10.1021/acs.jpcb.5c01826

**Published:** 2025-06-12

**Authors:** Amrutha Prabhakaran, Keshav Kumar Jha, Rengel Cane E. Sia, Mateusz Kogut, Jacek Czub, Julien Guthmuller, Colm Smith, Christopher S. Burke, Benjamin Dietzek-Ivanšić, Tia E. Keyes

**Affiliations:** † School of Chemical Sciences and National Centre for Sensor Research, 8818 Dublin City University, Dublin 9, Ireland; ‡ Research Department Functional Interfaces, Leibniz Institute of Photonic Technology Jena, Jena 07745, Germany; § Institute of Physical Chemistry and Abbe Center of Photonics, 9378 Friedrich Schiller University Jena, Jena 07743, Germany; ∥ Institute of Physics and Applied Computer Science, Faculty of Applied Physics and Mathematics, 49557 Gdańsk University of Technology, Narutowicza 11/12, Gdańsk 80233, Poland; ⊥ Department of Physical Chemistry, Gdańsk University of Technology, Narutowicza 11/12, Gdańsk 80233, Poland

## Abstract

Triplet–triplet annihilation upconversion (TTA-UC) implemented in liposomes may be a promising tool in drug delivery and sensing. Indeed, we recently demonstrated that colocalization of lipophilic reagents to the membrane hydrophobic core improves the TTA-UC efficiency in liposomes compared to solution. Here, we examined if the counter is true, i.e., we evaluate if TTA-UC is inhibited when the sensitizer and annihilator occupy different regions within a single leaflet of a liposome membrane. To test this hypothesis, we used a Ru­(II) complex, with tridentate ligand 2,6-di­(quinolin-8-yl)­pyridyl) (bqp) [Ru­(bqp)­(bpq-oct)]^2+^(Ru-bqp-oct) where oct is a C8 alkyl chain appended to facilitate integration into the liposome, as a sensitizer and diphenylanthracene (DPA) as an annihilator. TTA-UC from this pair was evaluated and compared in solution and liposomal nanovesicles. This Ru­(II)-bqp complex was selected for its exceptionally long-lived emission and high triplet quantum yield, due to its expanded N-Ru-N bite angles. In solution, TTA-UC was efficient with a quantum yield of 3.11%, but in liposomes, no anti-Stokes shifted emission was observed even with an increased concentration of sensitizer and annihilator in the membrane. Molecular dynamics simulations were used to understand this effect and confirmed poor co-orientation of sensitizer and annihilator in the membrane was responsible for lack of TTA-UC in the membrane. DPA was determined to orient at the hydrophobic core, while the cationic Ru complex is embedded shallowly at the membrane interface, the closest approach of donor and acceptor in the membrane was determined as 0.7 nm. This work highlights the critical importance of colocalization of sensitizers and annihilators, even within a single membrane leaflet to facilitate Dexter energy transfer through collision in membrane-constrained TTA-UC systems and the value of MD simulations in system design.

## Graphical abstract

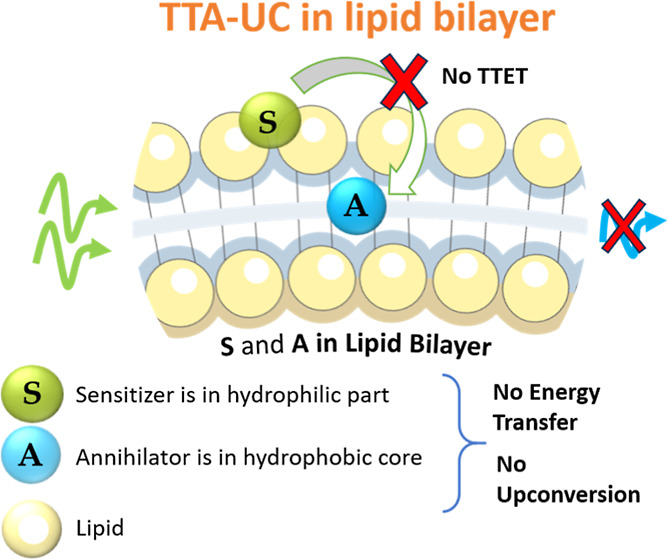

## 1 Introduction

Triplet–triplet annihilation upconversion (TTA-UC) is a photon upconversion process, that can operate under relatively low power excitation conditions and without the need for a coherent light source.^1–7^ It is thus garnering growing interest across diverse application domains from solar energy conversion to biomedical imaging and drug release.^8–10^

Implementation of TTA-UC in liposomal systems is of particular interest for the latter applications. There have been a number, though surprisingly few, studies to date on TTA-UC in liposomes, and they have shown that liposomal TTA-UC is typically highly effective due to the 2-dimensional concentration and confinement of the sensitizer and annihilator to the membrane.^11–14^ We recently demonstrated in a lipophilic BODIPY/Perylene system that two factors promote the liposomal TTA-UC efficiency; (i) membrane fluidity; we observed that in more fluidic membranes, collisional encounter between the sensitizer and the annihilator is enhanced, i.e., (ii) the co-orientation of the annihilator and the sensitizer; computation indicated that BODIPY and perylene both localized to the membrane hydrophobic core that led to the enhanced UC output compared to solution.^14^ To explore this further, we were interested in understanding in an efficient TTA-UC system in solution, if sensitizers and annihilators were confined to different parts of the membrane, this would compromise the TTA-UC output. To accomplish this, we combined a lipophilic annihilator with a charged Ru­(II) polypyridyl sensitizer and a lipophilic anchor.

Most effort to date in developing TTA-UC sensitizers has focused on porphyrins and metalloporphyrins since they offer advantages of high absorption cross section, often in the red and long-lived triplet state. Ru­(II) polypyridyl luminophores are also attractive sensitizers due to their efficient triplet state formation.^15–19^ For example, femtosecond transient absorption spectroscopy of well-known [Ru­(bpy)_3_]^2+^ (bpy = bipyridine) complex shows that intersystem crossing (ISC) is an ultrafast process occurring in 100 fs, from the first singlet excited state^20–22^ with triplet state formation occurring with near unity quantum efficiency.^23^

Castellano et al. reported one of the first examples of Ru­(II) polypyridyl TTA sensitization, using [Ru­(dmb)_3_]­[PF_6_]_2_ (where dmb is 4,4′-dimethyl-2,2′-bipyridine) as the sensitizer for TTA-UC with 9,10-diphenylanthracene (DPA) as the annihilator.^24^ The absorption maxima of the complex is at 450 nm, and the triplet excited state energy level was derived from the phosphorescence wavelength of 600 nm as 2.07 eV. Hence, DPA was used as the annihilator which has T_1_ excited state energy level of 1.77 eV (700 nm).^25,26^ Upconverted emission of DPA was observed at 430 nm under lower power density excitation at λ_ex_ = 514.5 nm, 24 mW or λ_ex_ = 532 nm, <5 mW.^27^ A qualitative Jablonski diagram of the TTA-UC process between the Ru­(II) sensitizer and the annihilator is shown in Scheme 1 that includes the calculated time-dependent density functional theory (TDDFT) energies of the states.

**Scheme 1:**
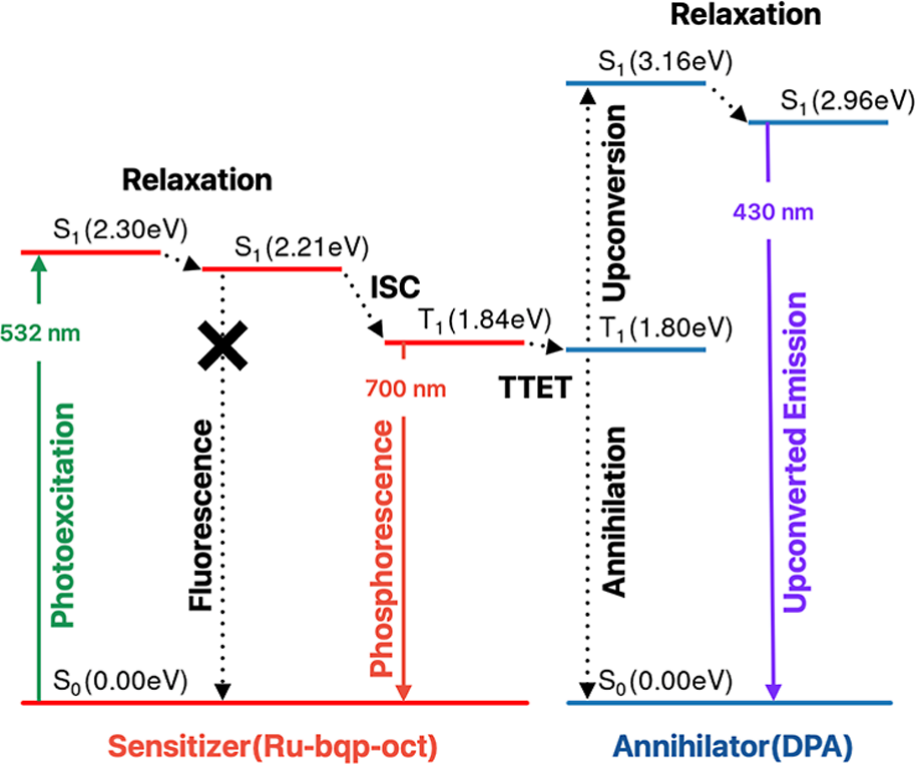
Schematic Illustration of the Jablonski Diagram of the TTA-UC Mechanism for the System Explored Here^a^

a ISC, intersystem crossing; TTET, triplet–triplet energy transfer. S_0_, ground state; S_1_, singlet MLCT of the sensitizer and singlet ππ* of the annihilator; T_1_, triplet MLCT of the sensitizer and triplet ππ* of the annihilator. The emission band observed for the sensitizer alone originates from the T_1_ excited state. The emission bands observed in the TTA process are the simultaneous sensitizer T_1_ phosphorescence emission and the annihilator S_1_ fluorescence emission. The calculated TDDFT energies are indicated in brackets.

Drawbacks of Ru­(II) polypyridyl complexes as triplet sensitizers are their typically blue to green (450–500 nm) excitation wavelengths and moderate molar absorption coefficient (ε) values. Since the excited state is ^3^MLCT, the heavy ruthenium atom tends to mix singlet and triplet character in the MLCT state; consequently, their triplet states are also shorter-lived than porphyrin sensitizers. Nonetheless, they are synthetically highly versatile, so readily tuned, and frequently highly photostable.^28–31^

Ru­(II) complexes with tridentate ligands with expanded N–Ru–N bite angles facilitate expansion to approximately octahedral geometry that dramatically extends triplet quantum yield and lifetime through the dual effects of rendering ^3^MC state less thermodynamically accessible and reducing the singlet character of the excited state.^32^

We thus applied a bis-tridentate Ru­(II) complex based on the bqp-ligand (bqp = 2,6-di­(quinolin-8-yl)­pyridyl) complex as a potential photosensitizer for TTA-UC in solution and liposomes. This complex originally reported by Abrahamsson et al.^33^ has the extended N–Ru–N bite angle, described above, it also shows preferred high extinction coefficient and red-shifted absorption compared to N,N coordinated Ru­(II) complexes, allowing excitation with green light.^32,34,35^ We demonstrated recently for a related complex, their suitability as TTA-UC sensitizers in polymeric systems.^18^ Here, we modified the (bqp = 2,6-di­(quinolin-8-yl)­pyridyl) with an octane tail to facilitate its integration into liposome Ru-bqp-oct ([Ru­(bqp)­(bqpCOOC_8_H_17_]­(PF_6_)_2_). The excited state lifetime of this complex is several microseconds long at room temperature, and we show that it is an efficient sensitizer for TTA-UC in solution, where we explore the thermodynamics and kinetics of the solution system using TDDFT calculations and transient spectroscopy. On extension to a liposomal system, however, in spite of effective integration of the sensitizer and the annihilator into the membrane, and across several concentration and annihilator iterations, we do not observe TTA-UC. Why this occurs is explored using molecular dynamics (MD) simulations.

## 2 Materials and Methods

### 2.1 Methods

1,2-Dioleoyl-*sn*-glycero-3-phosphocholine (DOPC) [purity (>99%)], and 1,2-dimyristoyl-*sn*-glycero-3-phosphocholine (DMPC) were purchased from Avanti Polar Lipids (Alabama, USA) and used without further purification. 1,2-distearoyl-*sn*-glycero-3-phosphoethanolamine-*N*-[amino­(polyethylene glycol (PEG))-2000] (DSPE-MPEG(2000) sodium salt) was purchased from Cayman chemical. 1,2-Dioleoyl-*sn*-glycero3-phosphoethanolamine-labeled ATTO655 (DOPE-ATTO655) was purchased from ATTO-TEC GmbH (Siegen, Germany). 9,10-DPA, anthracene, 9-anthracenecarboxylic acid, perylene, pyrene, phenanthrene, fluorescein, rubrene, sodium sulfite, d-(+)-glucose, sucrose, agarose, 1,4-dioxane, and phosphate buffer saline (PBS) tablets were purchased from Sigma-Aldrich (Wicklow, Ireland). Aqueous solutions were prepared using Milli-Q water (Millipore Corp., Bedford, USA). The polydimethylsiloxane (PDMS) silicon elastomer was purchased from Dow Corning GmbH (Wiesbaden, Germany) and mixed following supplier instructions. Monodisperse polystyrene (PS) latex sphere with a diameter of 4.61 ± 0.4 μm was obtained from Bangs Laboratories Inc. (Fishers, IN, USA).

### 2.2 Synthesis

Ru­(bqp)­(bqpCOOC_8_H_17_)]­(PF_6_)_2_ (Ru-bqp-oct) was analyzed. The asymmetric acid terminated parent complex [Ru­(bqp)­(bqpCOOH)]­(PF_6_)_2_, where bqp = 2,6-di­(quinolin-8-yl)­pyridyl was synthesized according to the procedure reported by Abrahamsson et al.^18,36^ Chemical structure of triplet sensitizer Ru-bqp-oct is given in Figure 1a.

**Figure 1:**
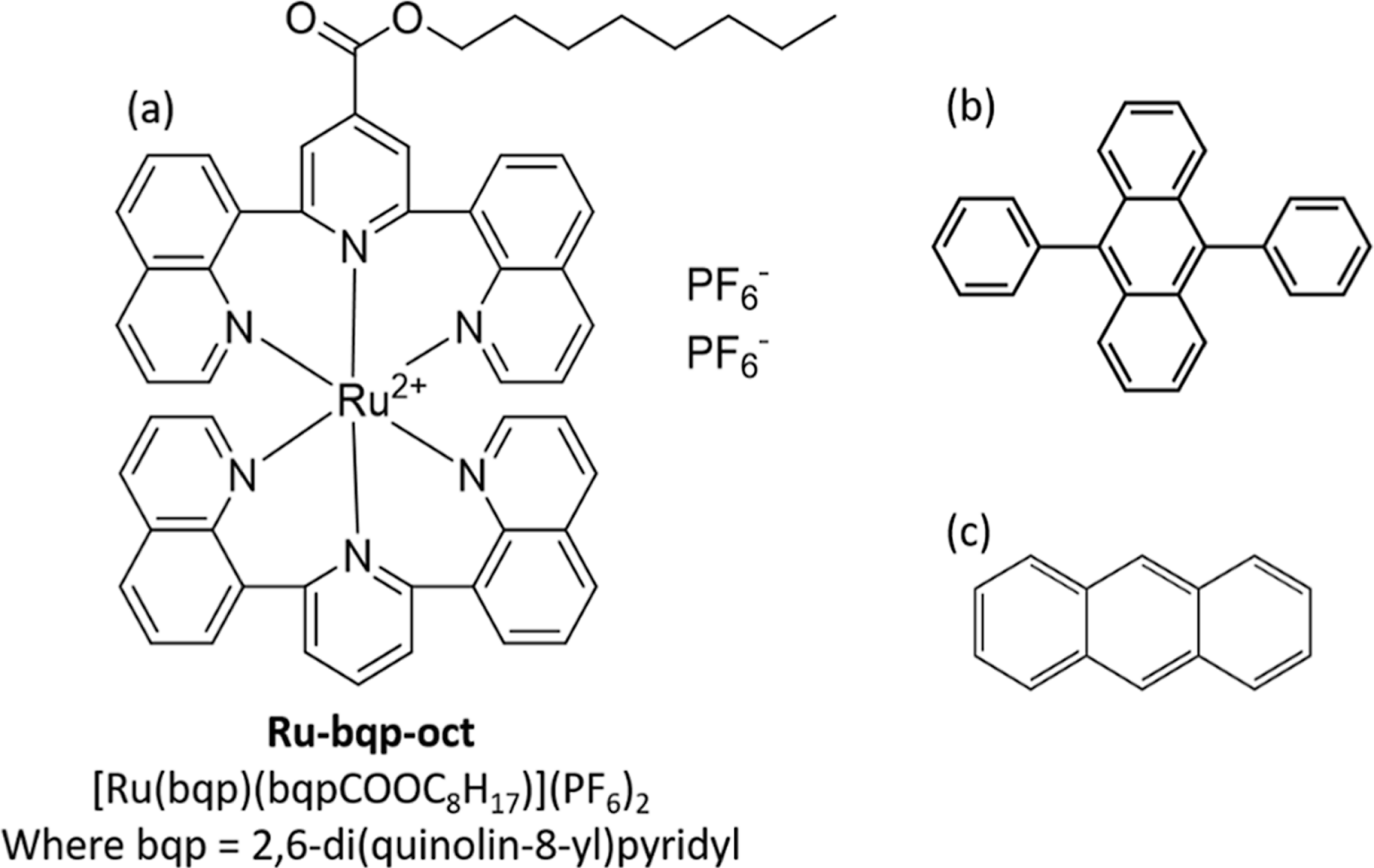
Chemical structures of (a) triplet sensitizer Ru-bqp-oct, and annihilators (b) 9,10-DPA and (c) anthracene.

Ru­(bqp)­(bqpCOOC_8_H_17_)]­(PF_6_)_2_, Ru-bqp-oct: Ru­(bqp)­(bqpCOOH)]­(PF_6_)_2_ (50 mg), was suspended in octanol (10 mL) and treated with five drops of concentrated sulfuric acid. The mixture was heated at reflux overnight, then cooled, and poured onto 100 mL of a NH_4_PF_6_ (aq.) solution. The Ru-octanol conjugate was extracted into dichloromethane (2 × 25 mL), and the organic extracts were combined and dried over anhydrous MgSO_4_, and then evaporated to obtain a crude red oil. The oil was twice subjected to column chromatography on silica gel using 98/2 CH_2_Cl_2_/CH_3_OH and 90/10/1 CH_3_CN/H_2_O/KPF_6_ (aq) as eluents. *R*_f_ ∼ 0.9. The product band was concentrated by rotary evaporation to precipitate a deep red residue that was filtered and dried. ^1^H NMR (600 MHz, (CD_3_)_2_CO) δ (ppm): 8.25–8.44 (m, 11H), 8.11 (d, 2H), 7.91–8.05 (m, 4H), 7.82–7.89 (m, 4H), 7.60 (m, 4H), 7.23 (m, 4H), 4.42 (m, 2H), 1.79 (m, 2H), 1.43 (m, 2H), 1.25–1.31 (m, 8H), 0.85 (t, 3H). HR-MS (ESI­(+)); found *m*/*z*, 462.1366 [M – 2PF_6_-]^2+^; calcd *m*/*z* for [C_55_H_46_N_6_O_2_Ru]^2+^, 462.1358.

### 2.3 Photophysical Measurements

Absorbance spectra were measured using a Jasco V670 spectrophotometer, and the data analysis was performed using Jasco Spectra Manager v2 software and OriginPro 2020b. Fluorescence and upconverted emission measurements were acquired using a Varian Cary Eclipse fluorescence spectrophotometer (Varian Cary Eclipse Software v1.1). Fluorescence lifetime measurements were accomplished on a PicoQuant FluoTime 100 Compact FLS time-correlated single photon counting (TCSPC) system using a 450 nm pulsed laser from PicoQuant PDL800-B source and an external Thurlby Thandar Instruments TGP110 10 MHz pulse generator to enable acquisition of long lifetime data. Data were collected up to 10,000 counts, and decay curves were achieved using PicoQuant Fluofit software and tail-fit statistical modeling. Fitting was evaluated with the tailfit criteria; 0.9 < χ^2^ < 1.1 and by analysis of the residuals.

### 2.4 Triplet–Triplet Annihilation Upconversion

A 10 mW fixed focus 532 nm laser (Edmund Optics) was used as the excitation source. Upconverted emission spectra were recorded in the biochemiluminescence mode and by blocking the excitation source from the fluorescence spectrophotometer. The laser power was measured using a power meter (Edmund Optics), and the diameter of the laser spot at 532 nm was 1 mm. Measurements were performed after deaeration by bubbling nitrogen through the samples for 30 min. All measurements were carried out in a quartz cuvette of 10 mm path length.

Threshold power density measurements: the solvent was deaerated using the freeze pump thaw method. Samples were prepared and transferred to 1 cm Schlenk cuvettes under an inert atmosphere for measurements. The emission spectra were recorded using an FLS980 emission spectrophotometer (Edinburgh Instruments) with a 532 nm (CW532–100, Roithner LaserTechnik GmbH) laser diode. A combination of neutral density filters (OD = 1 and/or 2) was used to record emissions at higher excitation powers.

### 2.5 Upconversion Quantum Yield Measurements

Upconversion quantum yields of deaerated samples (under N_2_) were measured on an Edinburgh Instruments FS5 spectrofluorimeter by using an integrating sphere. Briefly, the upconverted emission was collected, after which a neutral density filter (OD 3) was inserted to collect the excitation peak. Analysis was performed on Fluoracle software using the built-in sphere background correction.

### 2.6 Stern–Volmer Quenching Studies

Emission quenching studies were performed against 20 μM Ru-bqp-oct in acetonitrile, which was titrated with increasing concentrations of the quencher. Fluorescence emission spectra were measured for each quencher concentration. The Stern–Volmer quenching constant (*K*_sv_) and the bimolecular quenching constant (*k*_q_) were obtained by fitting the data according to the Stern–Volmer equation

1 $$\frac{I_{0}}{I}=\frac{\tau_{0}}{\tau }=1+K_{SV}\left[Q\right]$$

where *I*_0_ and *I* are the fluorescence emission intensities of the Ru-bqp-oct sensitizer in the absence and presence of quencher, respectively. τ_0_ and τ are the lifetimes of Ru-bqp-oct in the absence and presence of the quencher, respectively. [*Q*] is the molar concentration of the quencher. *K*_SV_ is the Stern–Volmer constant, *K*_SV_ = *k*_TTET_τ_0_. The lifetime of the Ru-bqp-oct sensitizer was obtained by fitting the luminescence intensity decays using a single exponential model for each concentration of the quencher separately. The slopes of the Stern–Volmer plots were obtained by fitting the plots to the linear equation in each case to obtain *K*_SV_ from which *k*_TTET_ was extracted for the entire range of quencher concentrations.

### 2.7 Time-Resolved Photophysical Studies

Nanosecond time-resolved absorption (ns-TA) spectra were acquired using a custom-built setup reported by Dura et al.^18,37^ The electronics and programming to record the difference absorption signal are developed by Pascher Instruments (Lund, Sweden). All ns-TA spectra were measured by placing the samples in quartz-made inert cuvettes of 1 cm path length. The pump pulse energy was 0.12 ± 0.01 mJ for all of the ns-TA measurements.

### 2.8 Preparation of Large Unilamellar Vesicles

The large unilamellar vesicles (LUV) (or liposomes) were prepared by the hydration-extrusion method.^38,39^ The lipid dissolved in chloroform was mixed with desirable concentrations of sensitizer and annihilators together or separately in a 1.5 mL vial. The solvent was evaporated by purging nitrogen, and complete evaporation of the solvent was ensured by putting the reaction vial under vacuum for 30–60 min. A thin lipid film was formed along the walls of the vial; this was hydrated with 1 mL of PBS and the lipid-dye mixture was thoroughly mixed by vortexing for 60 s. The resultant solution was taken in a 1 mL Hamiltonian syringe and extruded through a polycarbonate membrane of 100 nm pore size. The solution was passed through the membrane at least 11 times. For DMPC lipid, the extrusion was performed at 30 °C, which is above the melting transition temperature of DMPC. The hydrodynamic diameter of the resulting liposomes was measured using dynamic light scattering (DLS), Melvern Zetasizer Ultra.

### 2.9 Preparation of Giant Unilamellar Vesicles

The giant unilamellar vesicles (GUVs) were prepared by the electroformation method using a Nanion Vesicle Pro instrument.^40^ DOPC lipid was mixed together with Ru-bqp-oct, and the resulting solution was drop cast over the conductive side of indium–tin oxide slides within an O-ring of 0.7 mm thickness greased to the surface. The sample was dried using nitrogen flow, and the complete evaporation was ensured by placing the slides under vacuum for 45 min. Then, one slide was introduced to the Nanion instrument chamber, and warm 230 mM sucrose solution was then added inside the O-ring. On top of that slide, another slide was placed with the electric coils contacting the conductive side of both slides. Alternating current voltage of 0–3 V was applied at a temperature of 37 °C raised for 5 min, and the applied voltage 3 V was applied continuously at a frequency of 10 Hz for electroformation to take place. This process was continued for 170 min at the same temperature with falling time of 5 min at the end. After the completion of electroformation, the top slide was removed and the vesicle solution was collected with a pipette tip of enlarged size by cutting the edge. The vesicle solution was added to 230 mM glucose solution to uphold the osmolarity inside and outside the vesicles.

### 2.10 Confocal Microscopy of GUVs

The fluorescence images of the GUVs were collected using a Leica TSP inverted (DMi8) confocal microscope. A 40×-oil immersion objective was used with the excitation line chosen from a white light laser as an excitation source. The Ru-bqp-oct within the GUV was excited at 496 nm, and the emission was collected within 620–800 nm. Since the GUVs are highly mobile and difficult to focus during the imaging process, the sample was mixed with 0.5% agarose to restrict the movement of the vesicles and the images were taken after 10 min of agarose addition.

### 2.11 PDMS Microcavity Array Preparation

Hand-cleaved mica sheet pieces of 1 cm × 1 cm size were glued onto a glass slide, and then 20 μL of 0.1% solution of 4.61 μm sized PS spheres in ethanol was drop cast over the mica sheets. The PS spheres were self-assembled over the mica surface during the evaporation of ethanol. Once all the ethanol gets evaporated, a desirable hexagonal assembly of PS spheres was obtained. PDMS mixed with curing agent at 10:1 ratio was then added to the top of the glass slide without any air bubbles, and this was dried at 90 °C for about 60 min. The dried PDMS was removed from the glass slide, then sonicated for 15 min in THF to remove the PS spheres, and this resulted in the formation of microcavity arrays of approximately 2 μm diameter on the PDMS substrate. THF was evaporated by drying the substrate overnight. Since the PDMS substrate is hydrophobic in nature, it was oxygen plasma treated for 5 min to make it hydrophilic prior to lipid bilayer formation. The oxygen plasma treatment ensured aqueous filling inside the cavities and provided a sufficiently hydrophilic interface to support the lipid bilayers as the bilayers are unstable without this step. Prior to lipid monolayer transfer, the PDMS substrate was sonicated in PBS for 15 min to ensure the aqueous filling inside the cavities.^41^

### 2.12 Preparation of Microcavity-Supported Lipid Bilayers

The DOPC lipid bilayer was prepared over the aqueous filled microcavities, and this is called a microcavity-supported lipid bilayer (MSLB). The lipid monolayer was transferred onto the PDMS substrate using the Langmuir–Blodgett (LB) technique from the air–water interface.^42,43^ The lipid solution in chloroform of 1 mg/mL concentration was added dropwise to the water subphase of LB trough, and the solvent was allowed to evaporate for 10 min. The lipid monolayer was transferred to the PDMS substrate with microcavities at a surface pressure of 33 mN/m. Later the substrate was enclosed within a microfluidic chamber and liposomes doped with Ru-bqp-oct was introduced into the chamber. The sample was allowed to incubate for 90 min for lipid bilayer fusion, and any residual liposomes within the chamber was washed away by flushing with excess amount of PBS. The resultant MSLB was used for fluorescence correlation spectroscopic (FCS) and fluorescence lifetime imaging (FLIM) studies.

### 2.13 FLIM and FCS

Fluorescence lifetime imaging (FLIM) and fluorescence correlation spectroscopy (FCS) measurements on MSLB were performed using a MicroTime 200 system (PicoQuant GmbH, Germany) integrated with an FCS module, dual SPAD detection unit, TCSPC, and inverted microscope model Olympus X1–71 with an Olympus UPlan SApo 60*x*/1.2 water immersion objective. Further details are given in the Supporting Information.

### 2.14 Computational Studies

Quantum chemical calculations were performed with the program Gaussian 16.^44^ DFT was employed to calculate the geometry and the vibrational frequencies of the ground state (S_0_), while the excited-state properties (energy, geometry, vibrational frequencies) were obtained from TDDFT. Geometry optimizations and frequency calculations were performed using the def2-SVP basis set, while excited states energies and oscillator strengths were calculated using the larger def2-TZVP basis set. The B3LYP functional was employed for Ru-bqp-oct as B3LYP was shown to provide reasonable accuracy for MLCT transitions in Ru complexes.^45^ The MN15 functional was used for DPA because this functional provides improved accuracy over B3LYP for anthracene derivatives.^46^ Density functional dispersion corrections were included using the GD3BJ model.^47^ The properties of T_1_ were obtained using the Tamm-Dancoff approximation of the TDDFT because this approach is more consistent for triplet states than conventional TDDFT.^48^ The effects of the solvent (acetonitrile, ε = 35.6) were taken into account by the polarizable continuum model using the SMD solvation model. The vibrational frequency analysis confirmed that the obtained geometries are minima of the potential energy surface. The charge density difference was computed with the Multiwfn program.^49^

## 3 Theoretical Calculation–MD Simulations

### 3.1 Simulation Systems and MD Protocol

We used a DOPC lipid bilayer model, which was prepared using the CHARMM-GUI Membrane Builder.^50^ This bilayer consisted of 120 DOPC molecules and was embedded in a rectangular box measuring 6.4 × 6.4 × 7.8 nm. To solvate the bilayer, we added 5434 TIP3 water molecules.^51^ To maintain a physiological ionic strength of 0.15 M, 11 K^+^ and 13 Cl^–^ ions were included in each of the systems. Subsequently, the standard CHARMM-GUI energy minimization and equilibration protocols were applied. The lipids were modeled using the CHARMM36m force field,^52^ while the force field parameters for Ru-bqp-oct and DPA molecules were obtained from the CHARMM generalized force field (CGenFF)^53^ via the CHARMM-GUI server. To obtain a complete topology for Ru-bqp-oct, we used our in-house Gromologist library.^54^ The partial charges were determined by fitting to the electrostatic potential, which was calculated at the B3LYP-D3/def2svp level using Gaussian 16.^44^ The LJ parameters for the Ru^2+^ cation and the parameters for the N–Ru bonds and N–Ru–N angles were taken from the previous study.^55^ The complete sets of parameters used for Ru-bqp-oct and DPA can be found in the attached files named “Ru-bqp-oct.itp” and “DPA.itp”, respectively.

All energy minimizations and MD simulations were carried out using Gromacs 2020.^56^ The simulations were performed under periodic boundary conditions and in the *NPT* ensemble with the temperature kept at 310.15 K using the Nosé–Hoover thermostat^57^ with a coupling time of 1 ps, while the pressure was coupled semi-isotropically with a Parrinello–Rahman barostat at 1 bar with a coupling time of 5 ps.^58^ To calculate electrostatic interactions, the particle-mesh Ewald summation was applied with a real space cutoff equal to 1.2 nm and a Fourier grid spacing of 0.12 nm. van der Waals interactions were represented by the Lennard-Jones potential with a smooth cutoff with a switching radius of 1.0 nm and a cutoff radius of 1.2 nm. The P-LINCS algorithm^59^ was used to constrain the length of all bonds involving hydrogen atoms except the water molecules, for which SETTLE was used.^60^ The Verlet leapfrog algorithm was used to integrate the equations of motion with a time step of 2 fs.

### 3.2 Spontaneous Membrane Binding and Localization in the Membrane

To examine the preferred localization of the studied sensitizer and annihilator molecules within a lipid bilayer, we introduced single molecules of Ru-bqp-oct or DPA into the water phase of the pre-equilibrated DOPC system. Subsequently, each of the two systems thus prepared was subjected to MD simulation following the described protocol with three independent replicas conducted to ensure sufficient sampling. After the spontaneous penetration of the DPA molecules into the lipid phase, each replica was simulated for 1 μs. The distributions of molecules along the axis perpendicular to the membrane surface were computed after discarding the first 200 ns of each replica as equilibration.

### 3.3 Free-Energy Simulations for Membrane Penetration of Ru-bqp-oct

To study the sensitizer–membrane interactions, we determined how the system’s free energy changes as a function of the center-of-mass distance between the Ru-bpq moiety of the sensitizer molecule and the DOPC phosphate groups from both leaflets (see the inset in Figure S9. To this end, we performed replica exchange umbrella sampling (REUS) simulations using the PLUMED 2.8 plugin^61^ coupled to Gromacs. To capture the full penetration of the membrane by the Ru-bpq-oct sensitizer, the reaction coordinate was sampled in the range spanning from 0.0 nm (position of bilayer midplane) up to 4.0 nm (fully dissociated state) in 21 uniformly distributed and 0.2 nm-separated REUS windows. The initial configurations for these windows were obtained from the additional biased 0.5 μs long MD simulations, in which the sensitizer was pulled inside the membrane by a harmonic potential with a force constant of 480 kcal/(mol·nm^2^). In each of the REUS windows, the system was simulated for 1 μs, using the harmonic potential with a force constant of 240 kcal/(mol·nm^2^) to restrain the system along the reaction coordinate. The exchanges between neighboring windows were attempted every 2 ps, and the acceptance rate turned out to be ∼13%. The free-energy profiles were determined from the last 700 ns of the thus obtained trajectories using the standard weighted histogram analysis method (WHAM 2.0.9).^62^ Uncertainties were estimated using bootstrap error analysis, taking into account the correlation in the analyzed time series.

## 4 Results and Discussion

### 4.1 Photophysical Studies

Figure 2a shows the absorption (blue) and emission (red) spectra of the sensitizer Ru-bqp-oct obtained in acetonitrile, 10 μM. The absorption maxima and peak position of Ru-bqp-oct are consistent with those reported for [Ru­(dqp)_2_]^2+^ and parent complex previously without the oct tail.^18,63–65^ The absorption band centered at 343 nm is assigned to intraligand and interligand π–π* transitions, whereas the broad absorption band ranging from 400 to 600 nm with a maxima at 490 nm originates from a superposition of several singlet MLCT transitions (see Table S2).^33^ The π–π* transitions in the ligand moieties contribute to the absorption band in the UVB region.^66,67^

**Figure 2:**
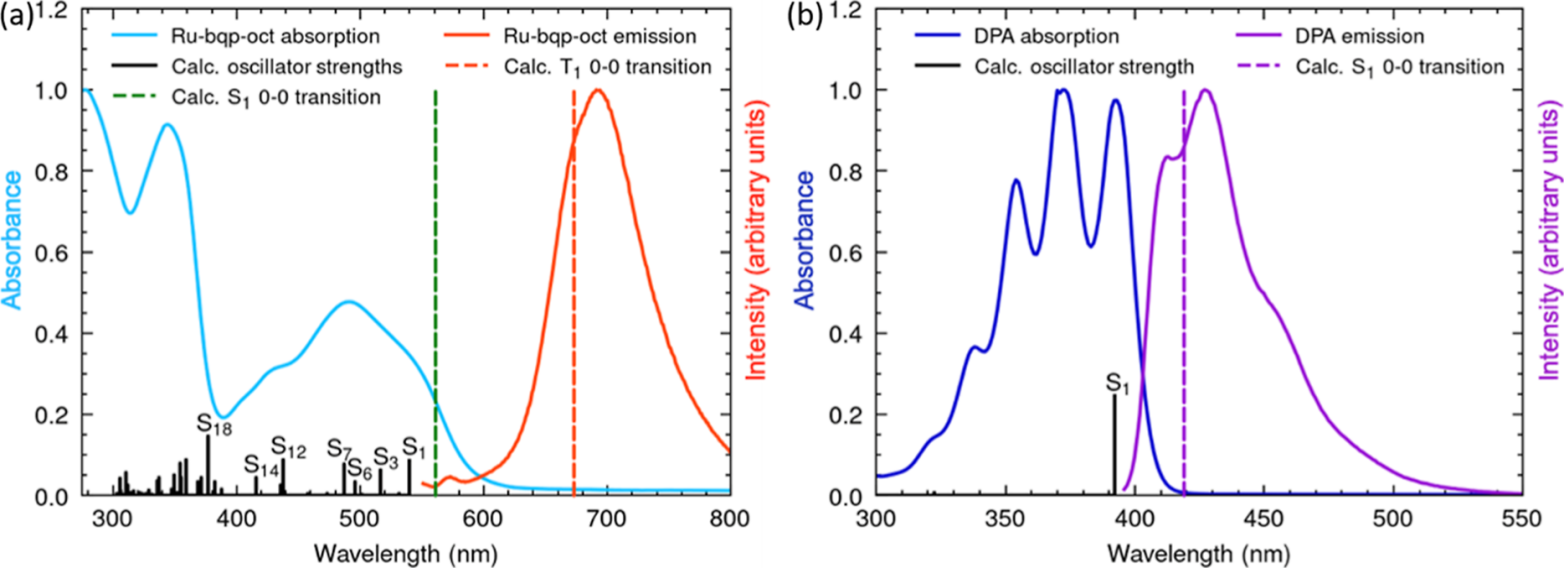
Normalized absorption and emission spectra of (a) 10 μM Ru-bqp-oct in acetonitrile and (b) 20 μM 9,10-DPA in acetonitrile. TDDFT vertical energies and oscillator strengths, S_1_ and T_1_ 0–0 transitions. The emission spectra of Ru-bqp-oct and DPA were collected by exciting at 490 and 395 nm, respectively. Excitation and emission slit width of 5 nm was used.

The emission spectrum of the complex in acetonitrile, under 490 nm excitation, shows a broad, intense emission centered at 690 nm, attributed to the lowest triplet MLCT state. The large Stokes shift, >∼200 nm, can be a key advantage in imaging or in TTA-UC where high concentrations do not lead to self-quenching. The emission lifetime of the Ru-bqp-oct complex decayed according to single exponential kinetics across all solvents explored. In acetonitrile, emission lifetime was recorded as 2.51 μs under N_2_ purge compared to 384 ns in air saturated solution. In acetonitrile, when deoxygenation was performed by freeze pump thaw (Figure S2), lifetime was recorded as 4.5 μs.^68,69^ Lifetime showed some solvent dependence; in dioxane, under N_2_ purge lifetime, it was recorded as 2.36 μs (714 ns aerated), and in dichloromethane, it was recorded as 2.1 μs (921 ns aerated). As expected in a useful triplet photosensitizer, the complex shows strong oxygen quenching.^70–72^ The absorption and emission spectra of the potential annihilator DPA in acetonitrile is given in Figure 2b.^73^ When compared, the emission profiles of both Ru-bqp-oct and DPA do not overlap.

### 4.2 Triplet–Triplet Annihilation Upconversion

Since this complex has a broad absorption extending to 600 nm, 532 nm was selected to excite TTA-UC as it coincides with the red edge of the MLCT band and does not directly excite DPA.

To optimize TTA-UC, the triplet sensitizer Ru-bqp-oct was evaluated for the TTA-UC output against several annihilators, including DPA, anthracene, 9-anthracenecarboxylic acid, phenanthrene, pyrene, perylene, fluorescein, and rubrene in deaerated acetonitrile. Upconversion was observed only with DPA or anthracene as the annihilator. Figure 3a shows representative upconverted emission from deaerated acetonitrile from Ru-bqp-oct as the sensitizer and DPA as the annihilator. This sensitizer–annihilator pair was then optimized across a range of ratios and 1:20, showed the best upconversion efficiency. Anthracene annihilator also provides comparable TTA-UC efficiencies, however, at low concentrations of Ru-bqp-oct (10 μM) and anthracene (200 μM), the efficiency of anthracene was found to be lower than DPA. The quantum yield for the anthracene-based system was measured as >0.1%, which is approximately 35 times lower than that of DPA. Nonetheless the anthracene offers upconverted signal that extends to the UV. The distinction in the efficiency likely originates from thermodynamic effects.

**Figure 3:**
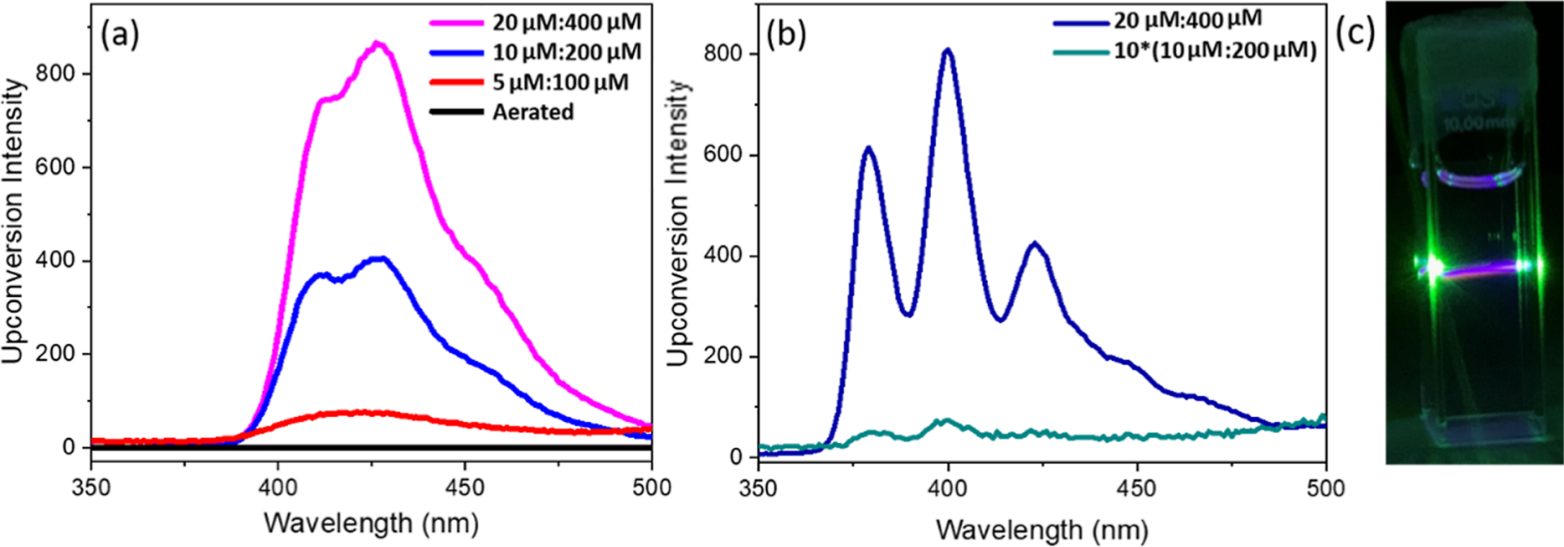
Upconverted emission from deaerated acetonitrile containing (a) Ru-bqp-oct and DPA at the 1:20 ratio with three different concentrations of 5 μM:100 μM, 10 μM:200 μM, and 20 μM:400 μM sensitizer/annihilator concentrations including one sample before deaeration showing the absence of TTA-UC and (b) 20 μM Ru-bqp-oct and 400 μM anthracene and 10 μM:200 μM (multiplied by 10). (c) Digital photograph showing the intense upconverted violet emission from deaerated acetonitrile containing 20 μM Ru-bqp-oct and 400 μM DPA. All samples are excited with a 532 nm green laser of 10 mW power, and the emission was collected at 5 nm slit width.

Focusing on the first pair, a strong oxygen sensitive violet emission was observed with the addition of DPA to the triplet photosensitizer Ru-bqp-oct. Controls confirmed that the anti-Stokes emission from the Ru-bqp-oct/DPA system is due to TTA-UC since the upconverted emission bands were absent for samples with DPA alone and Ru-bqp-oct alone under the same experimental conditions and also from the absence of an upconverted emission band in the presence of oxygen, as shown in Figure 3a (black). The upconverted emission from the deaerated acetonitrile solution containing Ru-bqp-oct and DPA is clearly visible by the eye, Figure 3c.

Solvent dependence of TTA-UC has been noted in other sensitizer/annihilator systems and linked to the solvent dependent triplet lifetime of the sensitizer; thus, we examined the persistence of TTA-UC across different solvents (acetonitrile, dichloromethane, ethyl acetate, dioxane, toluene, hexane, and CH_3_CN + water) maintaining Ru-bqp-oct: DPA ratio at 1:20 (20:400 μM sensitizer to annihilator). Interestingly, the process was solvent-dependent; only 1,4-dioxane and acetonitrile supported significant upconversion emission intensity, as shown in Figure S3. Upconversion quantum yield was measured as 3.11% [±0.05 in acetonitrile and 2.44% (±0.29) in dioxane] (the quantum yield in dioxane has relatively large standard deviation attributed to the lower solubility of the complex in dioxane). These are excellent values compared to other reported values for unsubstituted polypyridine ligands ruthenium complex.^74^ Higher values have been reported where substitution of the polypyridyl ligands with conjugated aryl groups extends *p*-delocalization, resulting in an excited state that is ^3^LC rather than ^3^MLCT reflected in extended emission lifetimes.^75,76^

### 4.3 Threshold Power Density Measurements

The upconversion intensity as a function of incident light power density at 532 nm excitation shows the characteristic profile of TTA-UC, with two linear regimes evident in Figure 4. Over the first, lower power regime, the upconversion intensity shows quadratic dependence on power density where TTA-UC is limited by the concentration of triplet annihilators available for collision. As incident power increases, a slope transition point, referred to as the power density threshold (*I*_th_) is reached, marking transition from the low annihilation regime to the high annihilation regime, where dependence of UC intensity is linear with power density.^73^ Beyond the *I*_th_, annihilator triplet has reached saturation concentration and maximum quantum efficiency is achieved, where upconverted emission is directly proportional to the number of triplet excited states sensitized.^18,77,78^

**Figure 4:**
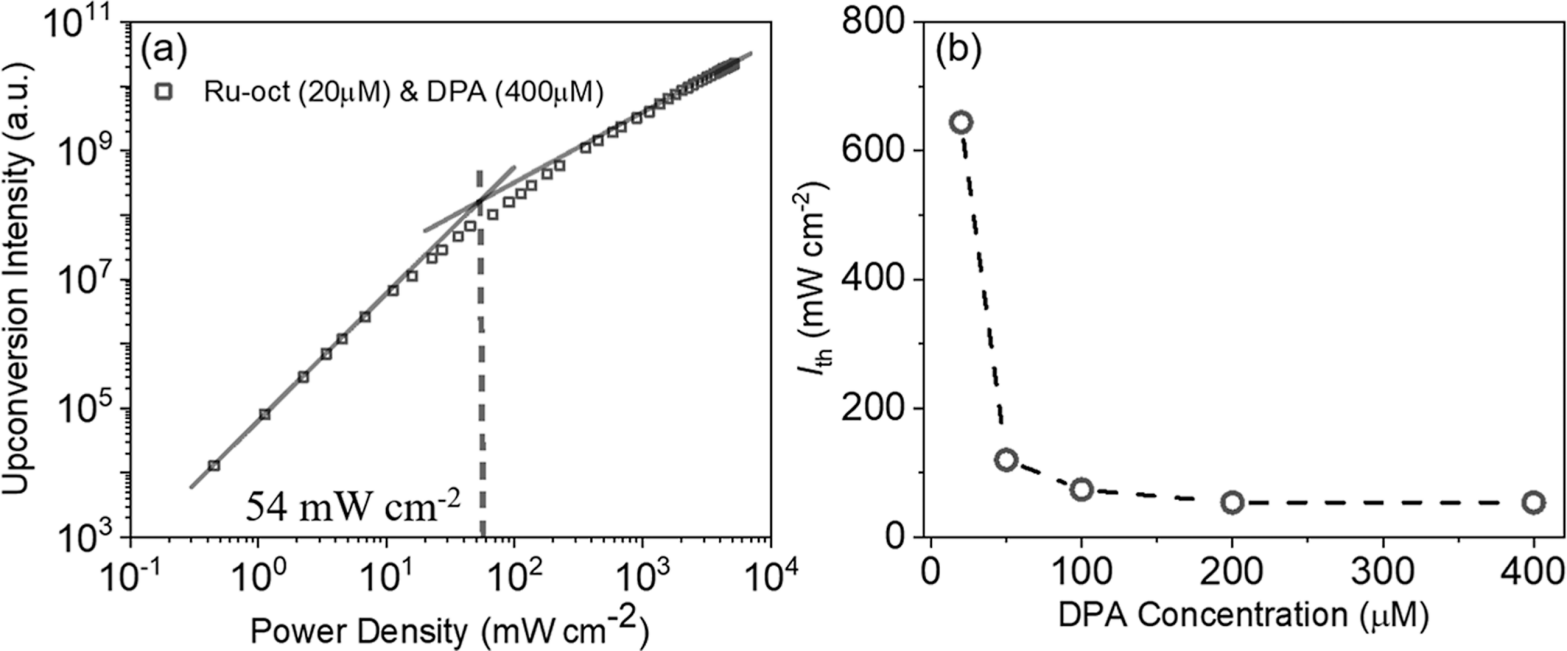
(a) Double logarithmic plot of integrated upconversion emission intensity measured as a function of the power of incident laser of 532 nm in a mixture of 20 μM Ru-bqp-oct and 400 μM DPA in deaerated acetonitrile. The linear fits with slopes 1 and 2 at high and low power regimes are included. *R* ^2^(COD) ≈ 0.98. (b) *I*_th_ vs DPA concentration.

The *I*_th_ value for 20 μM Ru-bqp-oct and 400 μM DPA is 54 mW cm^–2^ in acetonitrile. This low *I*_th_ value is an indication of high annihilation efficiency.^63,64^ The *I*_th_ values for 20 μM Ru-bqp-oct and 20 μM, 50 μM, 100 μM, and 200 μM DPA concentrations are 644 mW cm^–2^, 120 mW cm^–2^, 74 mW cm^–2^, and 54 mW cm^–2^, respectively, the figures are shown in Supporting Information (Figure S6). Figure 4b shows *I*_th_ vs DPA concentration, it is evident that *I*_th_ values decrease with the increasing DPA concentration, likely due to higher efficiency of energy transfer from triplet sensitizer to ground-state annihilator, see Supporting Information eqs S4 and S5 for details.^8,75^ However, excess annihilator concentration can result in back energy transfer from singlet annihilator to sensitizer,^75,79^ dimer formation,^10,18^ which along with diffusion limited triplet–triplet annihilation rate constant (*k*_TTA_) may limit lowering the *I*_th_ at high annihilator concentration (see eq S4).^75^ Therefore, an increase in the annihilator concentration from 200 to 400 μM does not reduce the *I*_th_ value below 54 mW cm^–2^. There are some reports on even lower *I*_th_ value, for example, Liang et al. has reported 3.63 mW cm^–2^ for an upconversion system containing Ru­(II) phenanthroline complex and 2-substituted anthracene.^75^

### 4.4 Nanosecond Transient Absorption Measurements

Nanosecond transient absorption studies of Ru-bqp-oct in the absence and presence of DPA are shown in Figure 5a,b, respectively. The ground state bleach (GSB) of Ru-bqp-oct (Figure 5a) is visible from ∼450 to 580 nm, the prominent excited state absorption (ESA) is evident from 380 to 420 nm, and stimulated emission (SE) is peaked at 700 nm; the kinetic traces from ESA and GSB region show lifetime of 4.2 μs. The transient absorption features are similar to other Ru-bqp-based molecules reported earlier.^18^ Upon addition of the annihilator, the Ru-bqp-oct (Figure 5b) shows similar GSB, ESA, and SE features, except from 400 to 500 nm, where additional features are evident due to formation of excited triplet DPA (^3^A*) species from the excited triplet sensitizer (^3^S*) through the triplet–triplet energy transfer (TTET) process. The decaying kinetic trace at 380 nm and growing kinetic trace at 450 nm are exactly the same (1.6 μs), which indicates the ESA of ^3^S* is decaying and simultaneously ESA of ^3^A* is building up through TTET. The lower lifetime of ^3^S* in the presence of DPA is attributed to quenching by the ground-state annihilator (^1^A) molecule. The ESA of ^3^A* decays through first order as well as bimolecular second-order decays.^40^ The quenching of Ru-bqp-oct in the presence of DPA is shown in Figure S8, the *K*_sv_ and *k*_TTET_ values are 1.49 × 10^4^ M^–1^ and *k*_TTET_ is 3.6 × 10^9^ M^–1^ s^–1^, respectively; which is in line with the literature reports.^18^ The bimolecular rate constant for annihilation of DPA molecules is calculated using mixed kinetic analysis, which provides the *k*_TTA_ value to be 3.5 × 10^9^ M^–1^ s^–1^, the details of the analysis and fit are provided in Supporting Information Figure S7. The high *k*_TTA_ rate constant is due to free diffusion of ^3^A* molecules generated by ^3^S* through the TTET process in the solution.

**Figure 5:**
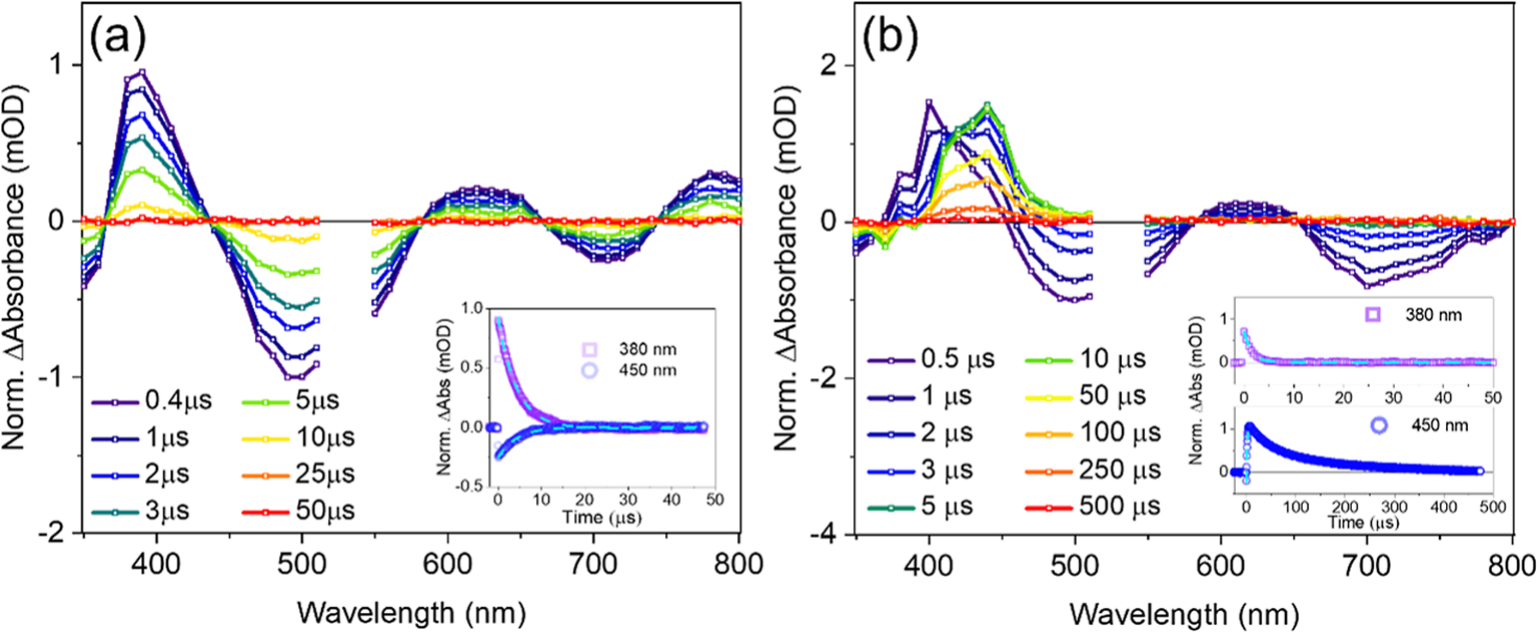
Normalized nanosecond transient absorption spectra of (a) Ru-bqp-oct, 20 μM in acetonitrile, inset: decay transient at 380 and 450 nm with monoexponential fits in dashed line, τ_dec_ at 380 nm = 4.2 μs; τ_dec_ at 450 nm = 4.2 μs. (b) Ru-bqp-oct 20 μM and DPA 100 μM. Inset: decay transient at 380 and 450 nm with monoexponential fits in dashed line. τ_dec_ at 380 nm = 1.6 μs; τ_inc_ at 450 nm = 1.6 μs.

### 4.5 Reconstitution of Ru-bqp-oct into the Lipid Bilayer

Given that efficient TTA-UC with a low *I*_th_ is observed in solution, we were interested to emulate this behavior within liposomes for potential biological applications. The aliphatic tail of Ru-bqp-oct facilitated its doping into DOPC liposomes that were prepared through the hydration-extrusion method described in the Experimental section.^80^ The resulting Ru doped liposomes were characterized by DLS, Figure S4a, which confirmed that homogeneous LUVs were formed with hydrodynamic radius of 145 ± 5 nm.

Since LUVs of this dimension are below the diffraction limit confocal microscopy to directly confirm reconstitution of Ru-bqp-oct within the bilayer of DOPC liposomes, in parallel, we created giant unilamellar vesicles of 5–10 μm diameter of the same lipid composition. The GUVs were prepared by electroformation^40,81^ and doped with 5 μm of Ru-bqp-oct. Confocal fluorescence imaging was carried out on the doped GUVs, exciting at 496 nm and monitoring between 620 and 800 nm to coincide with the ruthenium complex emission. A representative confocal image is shown in Figure S4b, where it is clear that the sensitizer is confined to the lipid bilayer of the membrane with no emission from the background solution. The lifetime of the ruthenium sensitizer within the lipid bilayer of the LUVs was measured as 703 ns (±2.5 ns) in aerated conditions and 2.09 μs (±0.03 μs) after purging with N_2_ for 30 min.

### 4.6 FCS and FLIM Studies of Ru-bqp-oct in the MSLB

To gain further confirmation that the Ru-bqp-oct is integrated into the liposome bilayer, we measured its diffusion coefficient, *D* which would be expected to reduce significantly on membrane inclusion.^82^ Measuring the translational diffusion using GUVs while feasible is challenging due to their high mobility as they tend to diffuse in and out of the confocal focus. This can be overcome by their inclusion in agarose gel, but this affects membrane fluidity and in turn the diffusivity of the probe. Instead, we exploited a lipid bilayer supported over an aqueous filled PDMS microcavity two-dimensional (2D) array platform. In this platform; microcavity supported lipid bilayer, MSLB, the lipid membrane is suspended over an aqueous filled pore so it retains the fluidity of liposome membrane but enables facile determination of the diffusion coefficient of lipid or reconstituted species such as lipid–protein, lipid-drug, and lipid–sensitizer interactions using FLCS.^83–86^

The fluorescence lifetime (FLIM) images collected from the DOPC bilayer doped with Ru-bqp-oct are shown in Figure 6. The reflectance image shown in Figure 6a is generated due to the back scattering of light from the aqueous filled cavities and, as described earlier, serves to identify the buffer-filled cavities over which the bilayer spans, where some cavities may remain unfilled, they are clearly distinguisable as they appear dark. Figure 6b shows an emission lifetime image of the same area exciting the Ru-bqp-oct. The DOPC lipid bilayer was colabeled with DOPE-ATTO655, a lipid probe well-established to embed in the bilayer, in order to confirm colocalization and facilitate focusing on the bilayer position. Figure 6c shows FLIM collected from the DOPE-ATTO655 dye excited with a 640 nm laser, that confirms the formation of the bilayer and that DOPE-ATTO655 colocalizes with Ru-bqp-oct.

**Figure 6:**
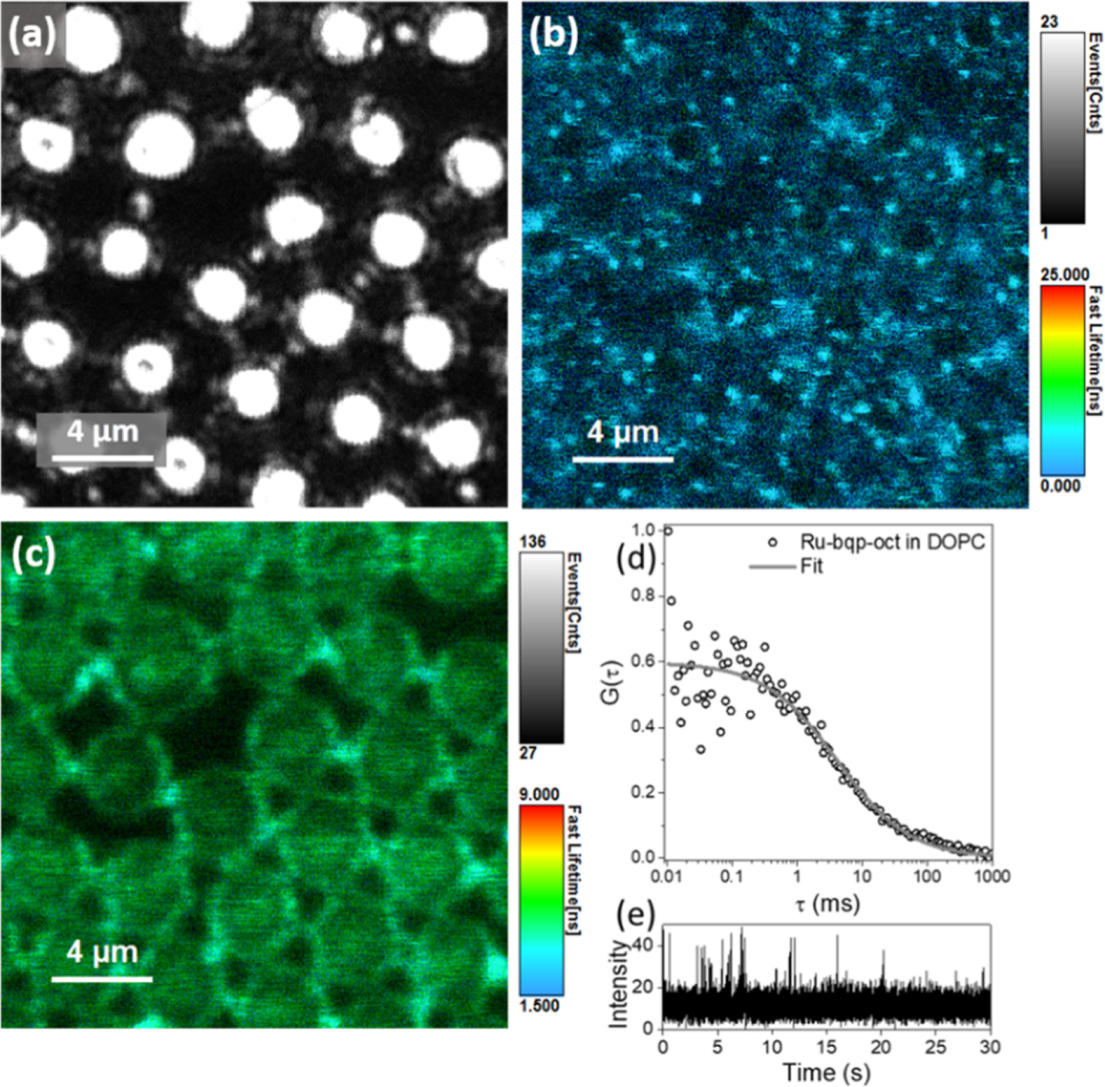
(a) Reflectance image of MSLB array showing the aqueous filled cavities, fluorescence lifetime images of the DOPC bilayer labeled with (b) 50 nM Ru-bqp-oct at 532 nm excitation and (c) DOPE-Atto655 at 640 nm excitation. Scale bar indicated 4 μm. (d) Representative FLCS autocorrelation function of DOPC MSLB labeled with 10 nM Ru-bqp-oct (upper leaflet) and the (e) corresponding intensity-time trace. FLCS was measured over 40–50 cavities, and the average is shown. The solid lines are the 2D diffusion fit using eq S1. All measurements were carried out in contact with PBS of pH 7.4.

FLCS was used to determine the translational diffusion coefficient of the sensitizer in the membrane. Due to the large cavity size, it is possible to carry out point FLCS measurements with the observation/detection volume at the center of the cavities with the spanned bilayer. The FLCS experiment on the MSLB platform and the measurements were repeated and averaged across many cavities (typically 40–50 cavities each time). A representative autocorrelation function (ACF) from FLCS is given in Figure 6d as *G*(τ) versus lag time (τ). The solid line is the fit to the 2D diffusion model, eq S2. The transit time was extracted from the fit, and the average diffusion coefficient was determined as 5.7 ± 2.2 μm^2^s^–1^, for Ru-bqp-oct according to eq S3. The diffusivity value for DOPC lipid labeled with DOPE-ATTO655 is 7.4 ± 1.4 μm^2^ s^–1^ with anomalous exponent value, α, determined as 0.871. The sensitizer, therefore, has a diffusivity value consistent with membrane interaction but interestingly, its α value, determined as 0.65, is significantly lower than 1, expected for purely Brownian motion. Such sub diffusion (α < 1) is not expected in a simple fluidic lipid membrane composed of DOPC so the anomalous diffusion and the associated spikes in the intensity-time trace shown in Figure 6e suggest there may be some aggregation of the complex in the membrane, possibly through the Oct tails. This may also explain the bright spots evident in the FLIM image of the Ru complex at the MSLB, although there is no evidence in the imaging of the GUV for this behavior, also, the concentration of the complex is lower in the MSLB.

### 4.7 TTA-UC in Model Membranes

After reconstitution was verified, we evaluated if TTA-UC could be observed in the lipid bilayer when Ru-bqp-oct is combined with the DPA annihilator. Initially, the studies were carried out using DOPC liposomes containing 0.5 μM Ru-bqp-oct and 10 μM DPA, but upon excitation at 532 nm under deaerated conditions in PBS, no upconverted signal was observed. Given the lifetime of the triplet state of the complex in membrane exceeds 2 μs, the diffusion coefficient of the complex and lipid/annihilator exceeds 5 μm^2^ s^–1^, vide infra. The Ru-bqp-oct triplet state is anticipated to be sufficiently long-lived to support TTA in the liposome. Indeed for an analogous sensitizer–annihilator pair in the polymer, efficient TTA-UC was observed and excited state electron transfer is also reported in liposomal systems containing Ru­(II) complexes.^18,87^ Nonetheless, even when we progressively increased the sensitizer concentration up to 10-fold (10×) to see if a TTA-UC signal could be generated, No Stokes-shifted signal was observed. Beyond 10×, the encapsulating efficiency within the liposomes was limiting, and precipitation of the complex from solution became evident.

The sensitizer to annihilator ratio was also varied from 1:20 to 1:5, 1:10, and 1:30, and extensive deaeration by both purging with inert gas, and adding sodium sulfite as an oxygen scavenger to remove molecular oxygen from the liposome solution was carried out, but no TTA-UC was observed. The study was also replicated across different lipid compositions, including as DMPC, DMPC/DSPE-PEG(2000), and DMPC/DMPE-PEG(2000) as PEG has been shown to be the optimal choice for obtaining sterically stabilized liposomes and also promotes liposome fluidty.^14,88^ Some representative spectra are given in Figure S5a, but across all attempts, in spite of strong TTA-UC in solution and with preconcentration of the sensitizer, which has been shown previously to promote TTA-UC in liposome, no TTA-UC was observed.

We recently demonstrated that colocalization and relative orientation of the sensitizer and annihilator in the membrane is critical for TTA-UC in liposomal systems.^14^ We propose that the present case is a negative case in point that further confirms this hypothesis. In the previous example, comprising a perylene annihilator with BODIPY charge transfer sensitizer that showed efficient TTA-UC from liposome, we demonstrated from calculation that the perylene and BODIPY sensitizer both embed deeply into the hydrophobic core of the lipid membrane where 2-D collision constrained to the membrane led to efficient Dexter energy transfer. Here, the ruthenium complex is assumed to be anchored to the membrane by its oct tail, but it is likely that the dicationic Ru orients at the membrane interface stabilized by the lipid phosphate head groups. Since energy transfer via the Dexter mechanism requires van der Waals sphere collision between sensitizer and annihilator, this may be impeded if the sensitizer and annihilator are localized at different parts of the membrane environment.

To test this hypothesis, we performed unrestrained atomistic MD simulations (see the Methods section for details), in which we observed that the DPA molecule, initially placed in an aqueous phase, rapidly (within tens of nanoseconds) penetrated into the core of a DOPC lipid bilayer. The equilibrium probability distribution along the bilayer normal extracted from the simulation (Figure 7) clearly shows that DPA binds to the membrane with high affinity, primarily residing in the center of its hydrocarbon core. In contrast, the Ru-bqp-oct molecule was not incorporated into the membrane. Within 1 μs of simulation, it bound to the membrane surface via the C8 alkyl chain that inserted into the bilayer (Figure 7). Since this simulation time might be too short to capture spontaneous membrane penetration by Ru-bqp-oct, we employed REUS simulations to determine the full free-energy profile of its interaction with the DOPC lipid bilayer. The resulting probability distribution, derived from the free energy profile by Boltzmann inversion (Figure 7), confirms that Ru-bqp prefers to remain on the membrane surface. The free-energy profile (Figure S9) reveals that its dissociation into the aqueous phase incurs an energetic cost of about 6 kcal/mol. Furthermore, insertion into the bilayer is even less favorable energetically (around 25 kcal/mol), with no free-energy minimum inside the membrane.

**Figure 7:**
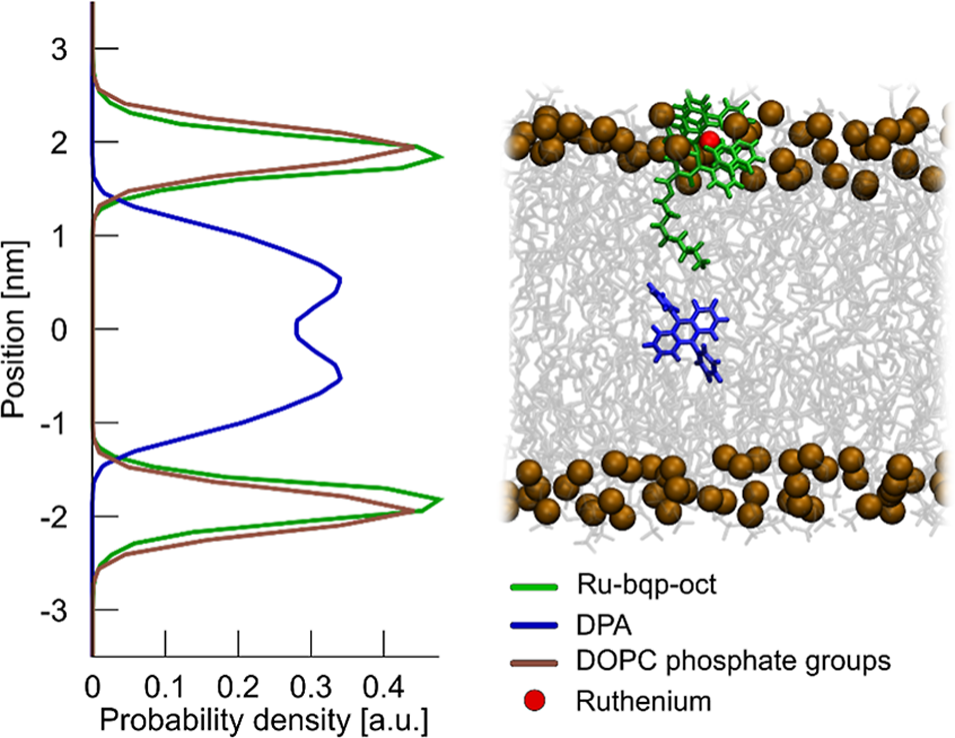
Preferred localization of Ru-bqp-oct and DPA molecules within a DOPC membrane. The probability distributions derived from our MD simulations show the positioning of the sensitizer and annihilator molecules along the axis perpendicular to the membrane surface. The brown curve represents the DOPC phosphate groups for reference. The 0.0 position corresponds to the bilayer midplane.

Overall, our simulation results suggest that the sensitizer and annihilator molecules localize in different regions of the membrane, showing no overlap in their distribution along the bilayer normal; our data indicate that the closest approach of sensitizer and annhilator in the membrane is 0.7 nm. This spatial separation prevents them from undergoing 2-dimensional collisions, explaining the lack of TTA-UC in our liposomal system.

## 5 Conclusions

In this study, the photophysics of TTA-UC from a sensitizer system comprising [Ru­(bqp)­(bqp-oct)]^2+^, where bqp is tridentate ligand 2,6-di­(quinolin-8-yl)­pyridyl and bqp-oct is the analogue with C8 tail, and a perylene acceptor were studied in detail in solution. The complex is an effective triplet sensitizer. At an optimized sensitizer/annihilator ratio of 1:20 in acetonitrile, the threshold power density (*I*_th_) was determined as 54 mW cm^–2^. Nanosecond transient absorption determined the triplet state lifetime of the sensitizer as 4.3 μs, which was quenched upon addition of the annihilator to <600 ns, and the TTET rate was determined as 1.6 μs. Given the efficiency of TTA-UC in solution, the sensitizer and annihilator were under reconstitution into DOPC liposomes. FCS was employed to study the diffusion of the sensitizer in the membrane using MSLB. The translational diffusion coefficient of the sensitizer was calculated to be 5.7 μm^2^ s^–1^ consistent with membrane integration and indicated sufficient mobility for sensitizer to annihilator collision to support TTA-UC in the membrane. Notably, in spite of extensive variation of sensitizer and annihilator concentration and ratio, no TTA-UC signal could be generated from the pair in liposomes. As previously reported, TTA-UC can be enhanced in liposomes but the colocalization of sensitizer and annihilator within the membrane for effective Dexter energy transfer is essential. MD simulations were carried out to understand the effect and demonstrated that the Ruthenium sensitizer is anchored to the membrane by the C8 tail but localized primarily to the aqueous membrane interface. The perylene annihilator by contrast localizes to the membrane core. The closest approach sensitizer and annihilator make toward each other is 0.7 nm which is apparently insufficient to support Dexter energy transfer. This study supports our recent report demonstrating the importance of colocalization of the sensitizer and annihilator to effective TTA-UC in membrane systems and highlights the value of applying MD simulations in understanding and designing membrane bound TTA-UC systems.

## Supplementary Material



## Notes

Associated spectra, equations, and calculations (PDF)

## #

Amrutha Prabhakaran—PASTEUR, Département de chimie, École normale supérieure, PSL University, 75005 Paris, France; Laboratoire Nanomédecine, Biologie extracellulaire, Intégratome et Innovations (NABI), Université Paris Cité, 45 rue des Saints-Pères, 75006 Paris, France. Keshav Kumar Jha—Material Science Division, Lawrence Berkeley National Laboratory, Berkeley, California 94720, USA. Christopher S. Burke—School of Chemistry and Analytical and Biological Chemistry Research Facility (ABCRF), University College Cork, T12 K8AF Cork, Ireland

## Abbreviations

TTA-UC: triplet–triplet annihilation upconversion
TTET: triplet–triplet energy transfer
Ru-bqp-oct: Ru­(bqp)­(bqpCOOC_8_H_17_)]­(PF_6_)_2_ where bqp = 2,6-di­(quinolin-8-yl)­pyridyl
DPA: diphenylanthracene
DOPC: 1,2-dioleoyl-*sn*-glycero-3-phosphocholine
DMPC: 1,2-dimyristoyl-*sn*-glycero-3-phosphocholine
DSPE-MPEG­(2000): 1,2-distearoyl-*sn*-glycero-3-phosphoethanolamine-*N*-[amino­(polyethylene glycol)-2000] sodium salt
FCS: fluorescence correlation spectroscopy
FLIM: fluorescence lifetime imaging microscopy
TCSPC: time-correlated single-photon counting
ns-TA: nanosecond time-resolved absorption spectroscopy
DOPE-ATTO655: 1,2-dioleoyl-*sn*-glycero3-phosphoethanolamine-labeled ATTO655
