# Supplementary material for: Triplet–Triplet Annihilation Upconversion Is Impeded in Liposomes that Prevent Sensitizer and Annihilator Co-Confinement
Source: J Phys Chem B. 2025 Jun 12;129(25):6220–32. doi: 10.1021/acs.jpcb.5c01826 (PMC12207582; doi:10.1021/acs.jpcb.5c01826)
Supplement: Supplementary file 1 [file jp5c01826_si_001.pdf]

ASSOCIATED CONTENT

Triplet-Triplet Annihilation Upconversion is Impeded in Liposomes that Prevent Sensitizer and Annihilator Co-Confinement.

Amrutha Prabhakaran,[1] Keshav Kumar Jha,[2,3] Rengel Cane E. Sia,[4] Mateusz Kogut,[5] Jacek Czub,[5] Julien Guthmuller,[4] Colm Smith[1] Christopher S. Burke,[1] Benjamin Dietzek-Ivanšić,[2,3] Tia E. Keyes[1*]

[1]School of Chemical Sciences and National Centre for Sensor Research, Dublin City University, Dublin 9, Ireland
[2]Research Department Functional Interfaces, Leibniz Institute of Photonic Technology Jena, Jena 07745, Germany
[3]Institute of Physical Chemistry and Abbe Center of Photonics, Friedrich Schiller University Jena, Jena 07743, Germany
[4]Institute of Physics and Applied Computer Science, Faculty of Applied Physics and Mathematics, Gdańsk University of Technology, Narutowicza 11/12, 80233, Gdańsk, Poland
[5]Department of Physical Chemistry, Gdańsk University of Technology, Narutowicza 11/12, 80233 Gdańsk, Poland

*Corresponding author

Email: tia.keyes@dcu.ie

Contents

1. Fluorescence lifetime imaging (FLIM) and correlation spectroscopy (FLCS)**Error! Bookmark not defined.**

2. Results..3

1. Fluorescence lifetime imaging (FLIM) and fluorescence correlation spectroscopy (FLCS)

In FLCS, the emission of the diffusing fluorophore through the detection volume is examined by calculating the auto-correlation curve, which assesses the self-similarity of the signal over time, as defined below.

$$G(\tau) = \frac{\langle\langle\partial I(t)\rangle\langle\partial I(t+\tau)\rangle\rangle}{\langle I(t)\rangle^2} \qquad \text{(S1)}$$

where ⟨⟩ denotes the time average, and ⟨∂I(t)⟩ and ⟨∂I(t+τ)⟩ are the fluorescent intensity fluctuations around the mean value at time, t and t+τ respectively, where τ is the lag time. The FLCS autocorrelation data were fitted to a 2D diffusion model using Equation S2:

$$G(\tau) = \left[\frac{1}{N}\right]\left[\frac{1}{1+\left(\frac{\tau}{\tau_D}\right)^{\alpha}}\right] \qquad \text{(S2)}$$

where G(τ) is the autocorrelation function of fluorescence fluctuations, N is the average number of diffusing fluorophores in the effective volume, τ is the delay time, τD is the diffusion time of the molecules across the confocal volume, α is the anomalous parameter, and c is the contribution of the diffusing species. The ACFs were fitted using a two-dimensional diffusion model to determine the diffusion time, and the diffusion coefficient was calculated using Equation S3:

$$D = \frac{\omega^2}{4\tau_D} \qquad \text{(S3)}$$

where D is the diffusion coefficient and ω is the 1/e2 radius of the confocal volume. ω was measured using ATTO-532/ATTO-655 (ATTO TEC, GmbH) dye solution of known diffusion coefficient at 20 ˚C in water. All measurements were performed with a dye concentration of 5 nM.

Fluorescence lifetime images were also captured using the same MicroTime 200 system. Each sample was recorded for 360 seconds at a resolution of 512 x 512. The data was analyzed using PicoQuant Symphotime software.

2. Results

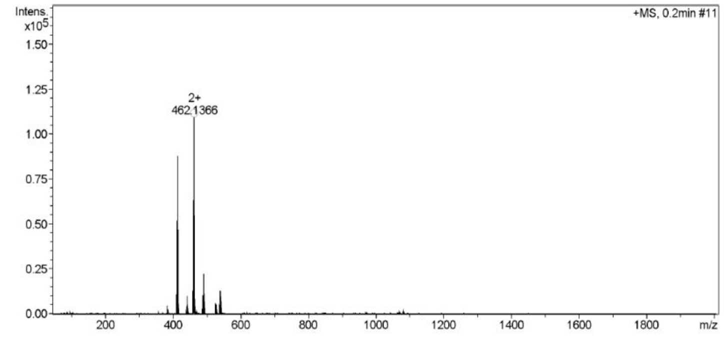

Meas. m/z	#	Ion Formula	m/z	err [mDa]	err [ppm]	rdb	N-Rule	e⁻ Conf	mSigma
462.136632	1	C55H46N6O2Ru	462.136487	0.1	0.3	36.5	-	odd	13.8

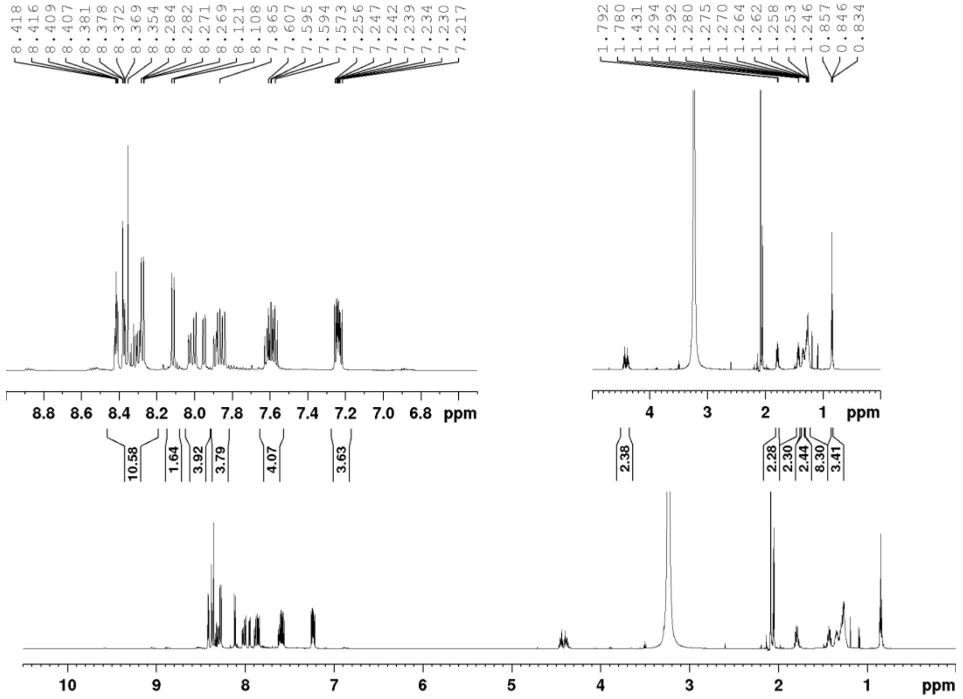

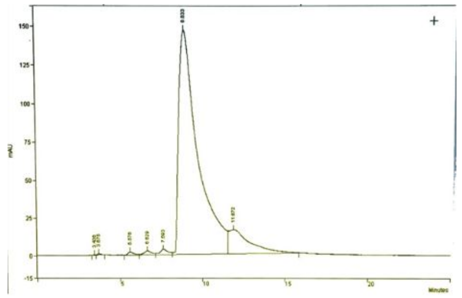

Figure S1: Structural characterisation data for Ru-bqp-Oct (A); HR-MS (ESI, MS+) spectrum of Ru-bqp-Oct with single mass analysis that matches the pre. (B) ^{1}H NMR spectrum (600 MHz, $(CD_3)_2CO$)) for Ru-bqp-oct, insets expand regions of interest for the Ru centre top left and the fac isomeric impurity tail top right. Integration is as expected for this aromatic, (metal complex) and octyl tail in aliphatic regions, a small amount (<3% of fac isomeric impurity) is evident in the baseline. Bottom, Reverse phase HPLC traces for Ru-bqp-acid (parent). Mobile phase: 75:25 water:acetonitrile with 0.1% TFA Hichrom C18 column, λ detection= 290 nm. The HPLC for the Ru-bqp-oct was not collected due to poor solubility in mobile phase, but evident as a single spot on TLC.

3.

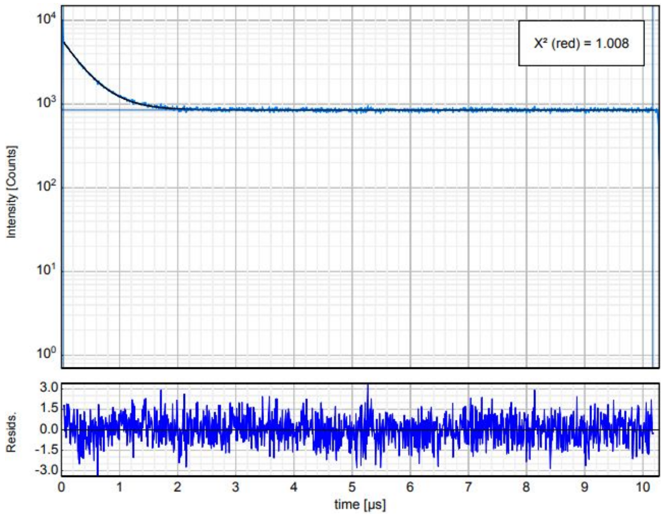

Figure S2. Lifetime decay curves and fit of 10 µM Ru-bqp-oct in acetonitrile under 450 nm excitation.

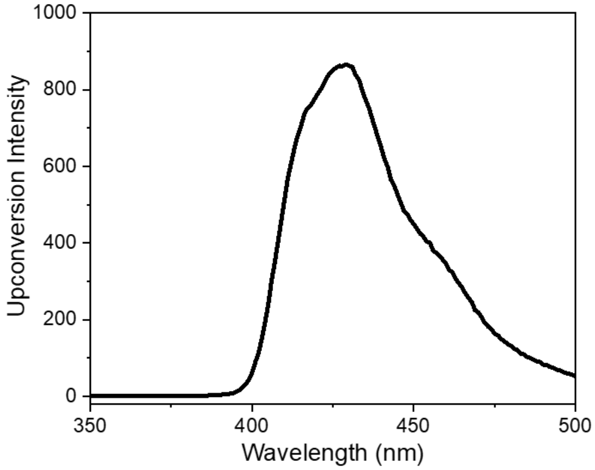

Figure S3. Upconverted emission from 20 µM Ru-bqp-oct and 400 µM DPC in deaerated 1,4-dioxane at 5 nm slit width under 532 nm excitation.

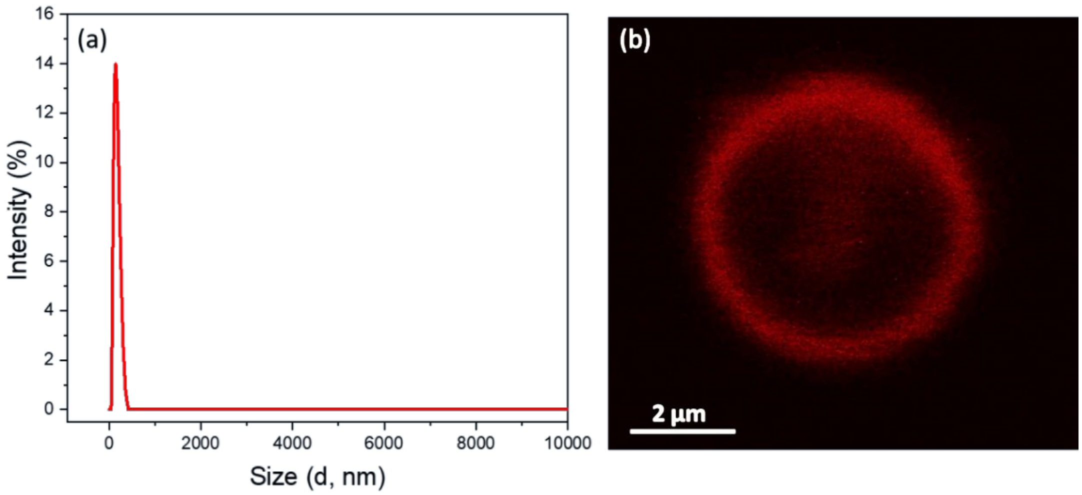

Figure S4. (a) DLS spectra of DOPC large unilamellar vesicles labelled with Ru-bqp-oct and (b) confocal image of GUV of DOPC lipid labelled with Ru-bqp-oct. λ_{ex} = 496 nm and the emission were collected within 620-800 nm.

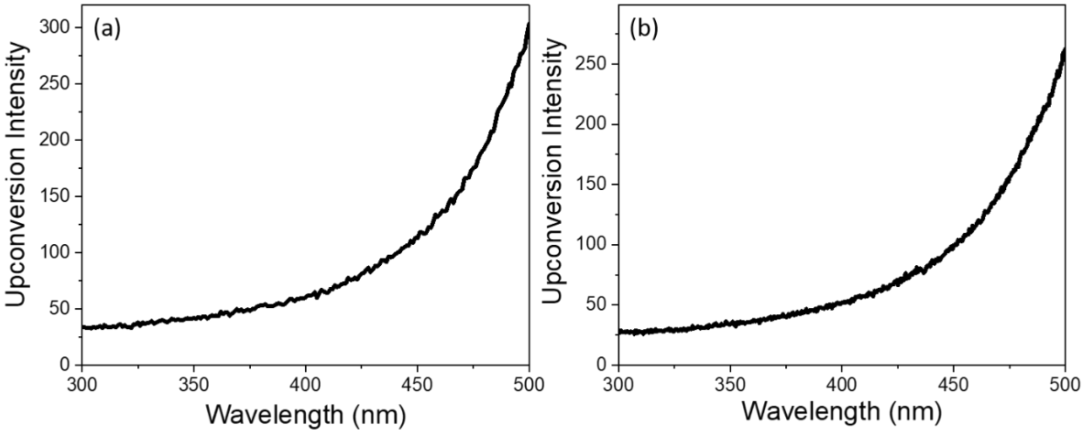

Figure S5. (a) DOPC and (b) DMPC:DSPE-MPEG(2000) liposomes of 140 nm diameter containing 5 µM Ru-bqp-oct and 100 µM DPA in deaerated PBS at 532 nm excitation.

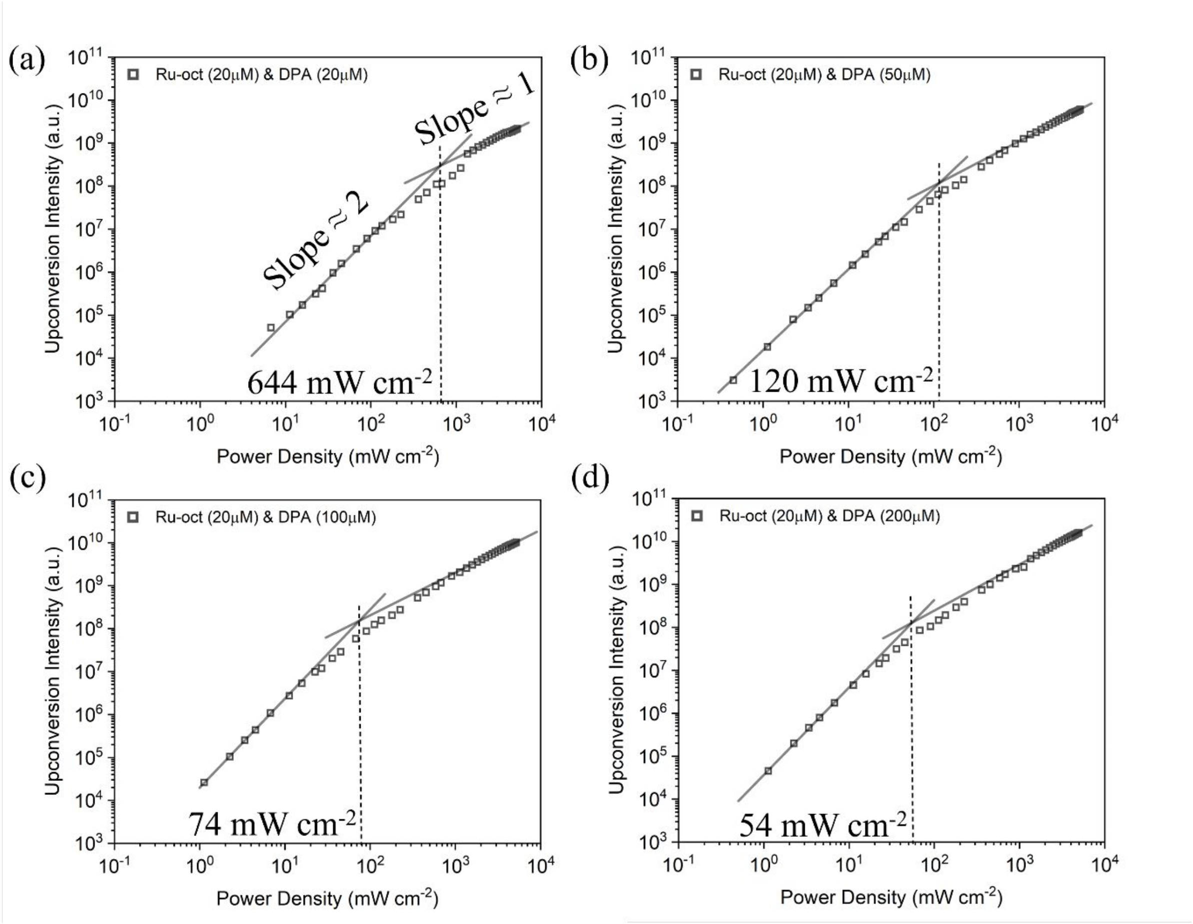

Figure S6. Double logarithmic plot of integrated upconversion emission intensity measured as a function of the power of incident laser of 532 nm in a mixture of 20 μM Ru-bqp-oct and (a) DPA 20 μM (b) DPA 50 μM (c) DPA 100 μM (d) DPA 200 μM DPA in deaerated acetonitrile. The linear fits with slopes 1 and 2 at high and low power regimes are included. R^2(COD) ≈ 0.98

The I_{th} can be described using below equation:

$$I_{th} = \frac{\left(k_T^A\right)^2}{2\phi_{TTET}\,\alpha[^1S]\,k_{TTA}} \quad \text{(S4)}$$

$$\phi_{TTET} = 1 - \frac{I}{I_0} \quad \text{(S5)}$$

k_T^A = first order decay rate of annihilator

ϕ_{TTET} = efficiency of energy transfer from triplet sensitizer to ground state annihilator

$\alpha[^1S]$ = absorption coefficient of sensitizer

k_{TTA} = second order decay rate constant of triplet-triplet annihilation

I = phosphorescence intensity of sensitizer in presence of annihilator

I_0 = phosphorescence intensity of sensitizer in absence of annihilator

The phosphorescence intensity of sensitizer (I) decreases in the presence of annihilator, increasing concentration of the annihilator causes higher quenching of the sensitizer; therefore, I decrease, which reflects in higher ϕ_{TTET}.

The rate of annihilation can be obtained using mixed kinetic analysis[1–4] given by equation S6 below:

$$\frac{[^3A^*]_t}{[^3A^*]_0} = \frac{e^{-k_T^A \cdot t}}{1 + [^3A^*]_0 \times \frac{k_{TTA}}{k_T^A} \times (1 - e^{-k_T^A \cdot t})} \qquad \text{Equation S6}$$

Where,

$[^3A^*]_t$	time dependent excited triplet concentration of the annihilator
$[^3A^*]_0$	initial excited triplet concentration of the annihilator

The transient absorption data at 450 nm is converted into excited triplet concentration of DPA ($^3A^*$), and fitted in to equation S6. The fit is shown in Figure S6. The fit reveals the values of

$[^3A^*]_0$ = 3.06 μM

k_T = 4×10^2 s^{-1}

k_{TTA} = 3.5×10^9 $M^{-1}s^{-1}$

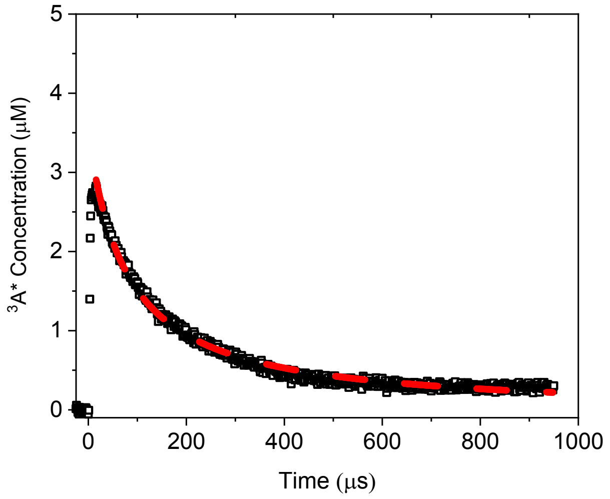

Figure S7. Mixed kinetic data analysis of DPA at 450 nm, dashed red line shows the fit of the data in black colour. R^2(COD) = 0.989 and reduced $\chi^2 \approx 3.16 \times 10^{-15}$.

The emission lifetime of Ru-bqp-oct in absence and presence of DPA is given below, which is used to calculate the Stern-Volmer plot. The lifetime was measured using nanosecond time-resolved emission spectroscopy and provided in Table S1.

Table S1. Ru-bqp-oct emission lifetime in absence and presence of DPA.

Ru-bqp-oct concentration (μM)	DPA concentration (μM)	Lifetime (μs)
20	0	4.2
20	20	3.2
20	50	2.5
20	100	1.9
20	200	1.1
20	400	0.6

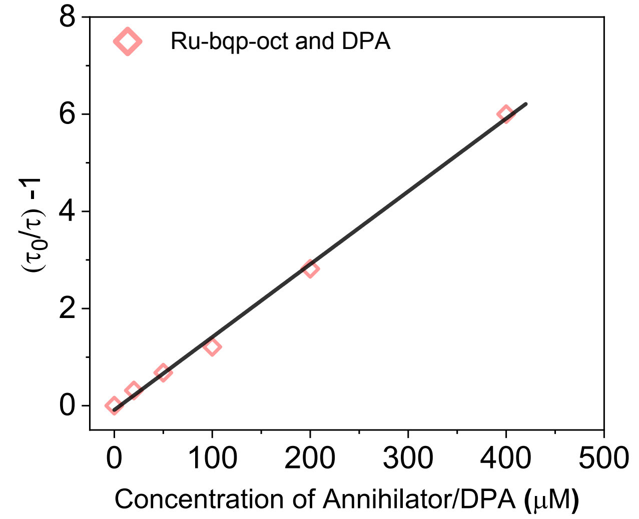

Figure S8. Triplet-triplet energy transfer rate constant (k_{TTET}) calculated using the dynamic Stern-Volmer equation. The obtained slope is $K_{SV} = 14990\pm406\ M^{-1}$, and R^2(COD) = 0.997.

The triplet lifetime is 4.2 μs (Table S1), the calculated k_{TTET} value using Equation 1 of the main text is $3.6x10^9\ M^{-1}s^{-1}$.

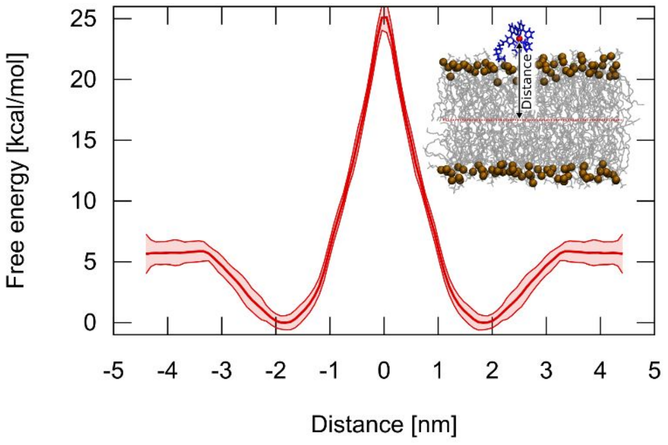

Figure S9. Free energy profile for the translocation of Ru-bqp-oct across a DOPC bilayer. The distance between the center of mass of the Ru-bpq moiety and the bilayer midplane is used as a reaction coordinate.

Table S2. Excited state properties of the Ru-bqp-oct complex at the S_0 geometry. The orbital transitions, weights, vertical absorption energies (VA), wavelengths (λ) and oscillator strengths (f) were calculated at the B3LYP/def2-TZVP, PCM=acetonitrile level of theory. Charge density difference (CDD) of the selected singlet states (hole: blue, electron: green).

State number	Transition	Weight (%)	VA (eV)	λ (nm)	f	CDD
S1	224 ->225	94	2.2972	539	0.087	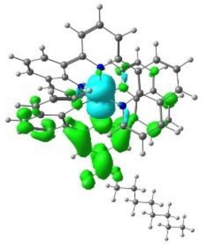
S3	224 ->226<br>223 ->225	71<br>20	2.4004	516	0.063	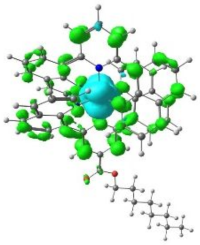
S6	224 ->228<br>223 ->227	76<br>14	2.5015	495	0.035	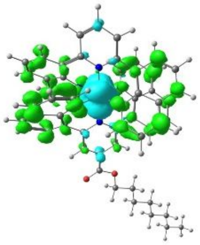
S7	223 ->227<br>224 ->228	65<br>16	2.5458	487	0.077	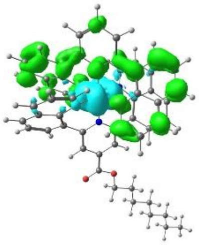
S12	222 ->228	90	2.8335	437	0.088	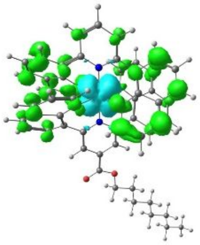

S14	223 ->229	93	2.9803	416	0.044	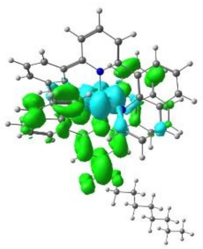
S18	221 ->225	70	3.2868	377	0.146	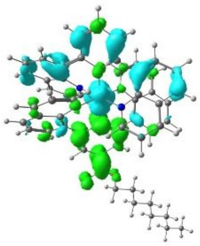

Table S3. Charge density difference of the S_1 and T_1 states of Ru-bqp-oct and DPA (hole: blue, electron: green).

S_1	T_1
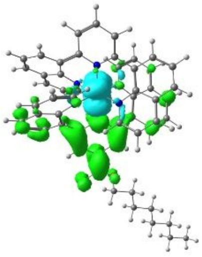	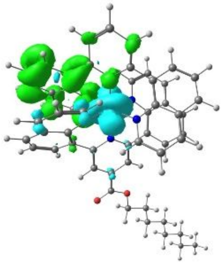
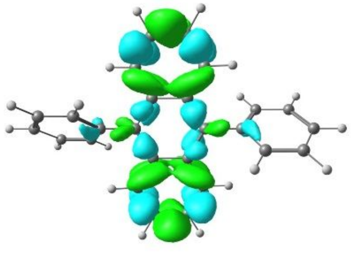	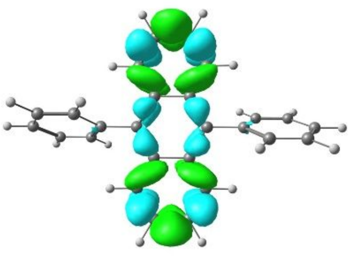
